# Examination of soluble integrin resistant mutants of foot-and-mouth disease virus

**DOI:** 10.1186/1743-422X-10-2

**Published:** 2013-01-02

**Authors:** Paul Lawrence, Michael LaRocco, Barry Baxt, Elizabeth Rieder

**Affiliations:** 1 Foreign Animal Disease Research Unit, United States Department of Agriculture, Agricultural Research Service, Plum Island Animal Disease Center, PO Box 848, Greenport, NY, 11944-0848, USA

**Keywords:** Soluble receptor, α_v_-integrin heterodimers, Foot-and-mouth disease virus (FMDV)

## Abstract

**Background:**

Foot-and-mouth disease virus (FMDV) initiates infection via recognition of one of at least four cell-surface integrin molecules α_v_β_1_, α_v_β_3_, α_v_β_6_, or α_v_β_8_ by a highly conserved Arg-Gly-Asp (RGD) amino acid sequence motif located in the G-H loop of VP1. Within the animal host, the α_v_β_6_ interaction is believed to be the most relevant. Sub-neutralizing levels of soluble secreted α_v_β_6_ (ssα_v_β_6_) was used as a selective pressure during passages *in vitro* to explore the plasticity of that interaction.

**Results:**

Genetically stable soluble integrin resistant (SIR) FMDV mutants derived from A24 Cruzeiro were selected after just 3 passages in cell culture in the presence of sub-neutralizing levels of ssα_v_β_6_. SIR mutants were characterized by: replication on selective cell lines, plaque morphology, relative sensitivity to ssα_v_β_6_ neutralization, relative ability to utilize α_v_β_6_ for infection, as well as sequence and structural changes. All SIR mutants maintained an affinity for α_v_β_6_. Some developed the ability to attach to cells expressing heparan sulfate (HS) proteoglycan, while others appear to have developed affinity for a still unknown third receptor. Two classes of SIR mutants were selected that were highly or moderately resistant to neutralization by ssα_v_β_6_. Highly resistant mutants displayed a G145D substitution (RGD to RDD), while moderately resistant viruses exhibited a L150P/R substitution at the conserved RGD + 4 position. VP1 G-H loop homology models for the A-type SIR mutants illustrated potential structural changes within the integrin-binding motif by these 2 groups of mutations. Treatment of O1 Campos with ssα_v_β_6_ resulted in 3 SIR mutants with a positively charged VP3 mutation allowing for HS binding.

**Conclusions:**

These findings illustrate how FMDV particles rapidly gain resistance to soluble receptor prophylactic measures *in vitro*. Two different serotypes developed distinct capsid mutations to circumvent the presence of sub-neutralizing levels of the soluble cognate receptor, all of which resulted in a modified receptor tropism that expanded the cell types susceptible to FMDV. The identification of some of these adaptive mutations in known FMDV isolates suggests these findings have implications beyond the cell culture system explored in these studies.

## Introduction

Foot-and-mouth disease virus (FMDV) is responsible for the most economically important viral disease of cattle and other cloven-hoofed animals [1-5]. FMDV, the prototypic member of the *Aphthovirus* genus of *Picornaviridae,* utilizes *in vitro* four integrin heterodimers (α_v_β_1_, α_v_β_3_, α_v_β_6_, and α_v_β_8_) for attachment to host cells and entry via clathrin-coated pits (CCPs) [6-14]. A prominent surface-exposed loop connecting the βG-βH strands (G-H loop) of the VP1 capsid protein contains a highly conserved Arg-Gly-Asp (RGD) motif, a recognition sequence for the α_v_-integrin family of cell surface receptors [6,15-17]. Limited trypsin proteolysis removes the G-H loop, producing FMDV particles considerably less infectious relative to untreated virions, highlighting the importance of this region for productive infection [18-20]. Following integrin binding, CCPs internalize virus into acidic endosomes where uncoating occurs. FMDV field isolates continually passaged in cell culture adapt to utilize heparan sulfate (HS) as an alternative receptor, and exhibit attenuated pathogenicity [21-24].

Previously, soluble α_v_β_3_ and α_v_β_6_ lacking the transmembrane and cytoplasmic tail domains were shown to still function as FMDV receptors [25]. Pre-treatment of serotype A and O FMDV particles with soluble secreted bovine α_v_β_3_ and α_v_β_6_ prior to application on permissive cell lines was investigated as an antiviral therapy. Interestingly, only soluble α_v_β_6_ limited FMDV attachment to host cells by competing for receptor binding sites on virus particles. Soluble α_v_β_3_ exhibited a low affinity interaction with the virus particles and failed to attach to FMDV in the same manner as α_v_β_6_ with no significant effect on infectivity. It remains to be determined whether binding to blocking molecules such as soluble receptor, will impact FMDV interaction with the cell-membrane receptor or affect viral growth.

**Figure 1:**
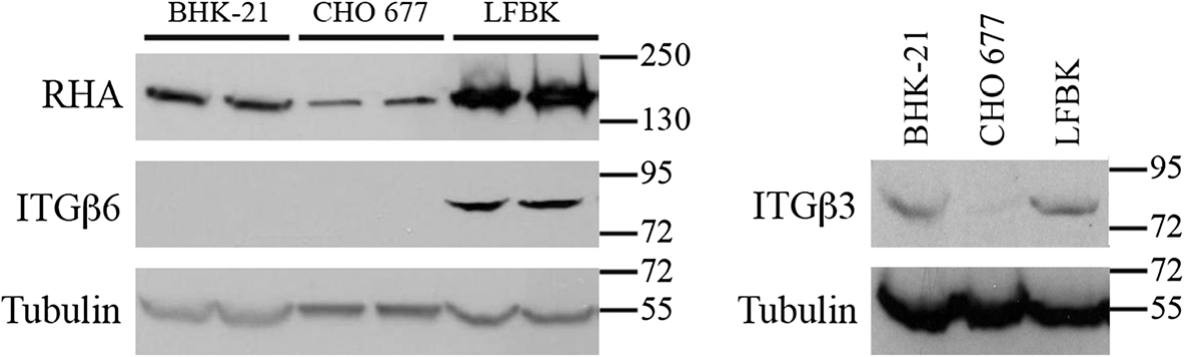
**Integrin expression profile.** BHK-21, CHO 677, and LFBK cell lysates were examined by Western blot probing with: anti-β_3_ integrin (ITGβ3), anti-β_6_ integrin (ITGβ6), and anti-RHA. Equivalent loading between lanes was confirmed by probing with anti-tubulin-α. Molecular weight ladder bands are indicated.

Here, we conducted *in vitro* experiments to evaluate the selective pressure exerted by soluble receptor protein on FMDV attachment and examined the evolution of virus-host cell interactions. Studies conducted with poliovirus and its cognate receptor [26,27] showed that surface and internal capsid residues regulate attachment to the receptor and conformational change of the virus. Here, sub-neutralizing levels of soluble secreted bovine α_v_β_6_ (ssα_v_β_6_) were used to develop soluble receptor resistant mutants of FMDV A24 Cruzeiro. Of the 4 α_v_-integrins used by FMDV for host cell attachment, the β_6_ heterodimer was selected on the basis that ssα_v_β_6_ most substantially impeded FMDV infection [25]. Additionally, the β_6_ heterodimer was shown to be most responsible for the tissue tropism of FMDV in cattle [28]. Following 3 successive passages of FMDV (serotype A and serotype O) pre-treated and co-incubated with ssα_v_β_6_ on the LFBK cell line, which is permissive to infection by all 7 serotypes of FMDV (Figure 1, Table 1) [29], virus was isolated that persisted despite the presence of soluble integrin (SI). The A-type isolates exhibited mutations in the normally conserved RGD motif or just outside of it within the G-H loop of VP1, while the O-type mutants displayed changes in VP3 and residues proximal to the VP1 RGD motif. SI resistant (SIR) FMDV mutants were further characterized for altered receptor tropism, relative sensitivity to neutralization by SI, as well as sequence and structural alterations in the G-H loop.

**Table 1:** Receptor repertoire of tested cell lines

**Cell Line**	**αvβ1**	**αvβ3**	**αvβ6**	**αvβ8**	**HS**	**Reference**
LFBK	+	+	+	*	+	Figure 1 (this paper); [29]
CHO K1	-	-	-	-	+	[30-37]
CHO 677	-	-	-	-	-	[21,30,38-41]
IBRS2	-	-	-	+	-	[16,42]
COS-1	*	-	-	-	+	Figures 3 and 6 (this paper); [7,36,37]

^+^ Confirmed expression of the receptor molecule on the cell line indicated.

^-^ Confirmed lack of expression of the receptor molecule on the cell line indicated.

^*^ Unclear if the receptor molecule is expressed on the cell line indicated.

The receptor profile of α_v_β_1_, α_v_β_3_, α_v_β_6_, α_v_β_8_, and HS on 6 cell lines used was tabulated with references to studies that directly demonstrated the receptor profile or did so indirectly by virus growth profiles.

## Results

### Selection of FMDV serotype A SIR mutants

To examine the adaptability of serotype A FMDV (represented by A24 Cruzeiro) to the presence of soluble receptor during infection, we employed ssα_v_β_6_, which is preferentially exploited by serotype A FMDV for host cell attachment [7,25]. As previously described, ectodomains of α_v_ and β_6_ integrin subunits were secreted from stably transfected cells, which were properly folded and capable of heterodimerization (data not shown) [25]. The bovine kidney LFBK cell line was selected for these studies on the basis that it is susceptible to infection by all 7 FMDV serotypes, similar to primary bovine kidney cell culture and [29]. Western blot analysis confirmed that LFBK cells express both β_3_ and β_6_ integrins (Figure 1, Table 1). As expected, CHO 677 cells [30,38] did not express β_3_ and β_6_ integrins [21,30-33,39-41]. While BHK-21 cells expressed β_3_ integrin, we were unable to detect the presence of β_6_ integrin. As an additional control, RNA Helicase A (RHA), known to be expressed in all 3 cell lines, was also detected. By extension, these findings allowed for the potential that both α_v_β_3_ and α_v_β_6_ could be found on the surface of LFBK cells, which is consistent with repeated experiments showing the susceptibility of this cell line to non-HS-adapted field isolates of FMDV (data not shown).

**Figure 2:**
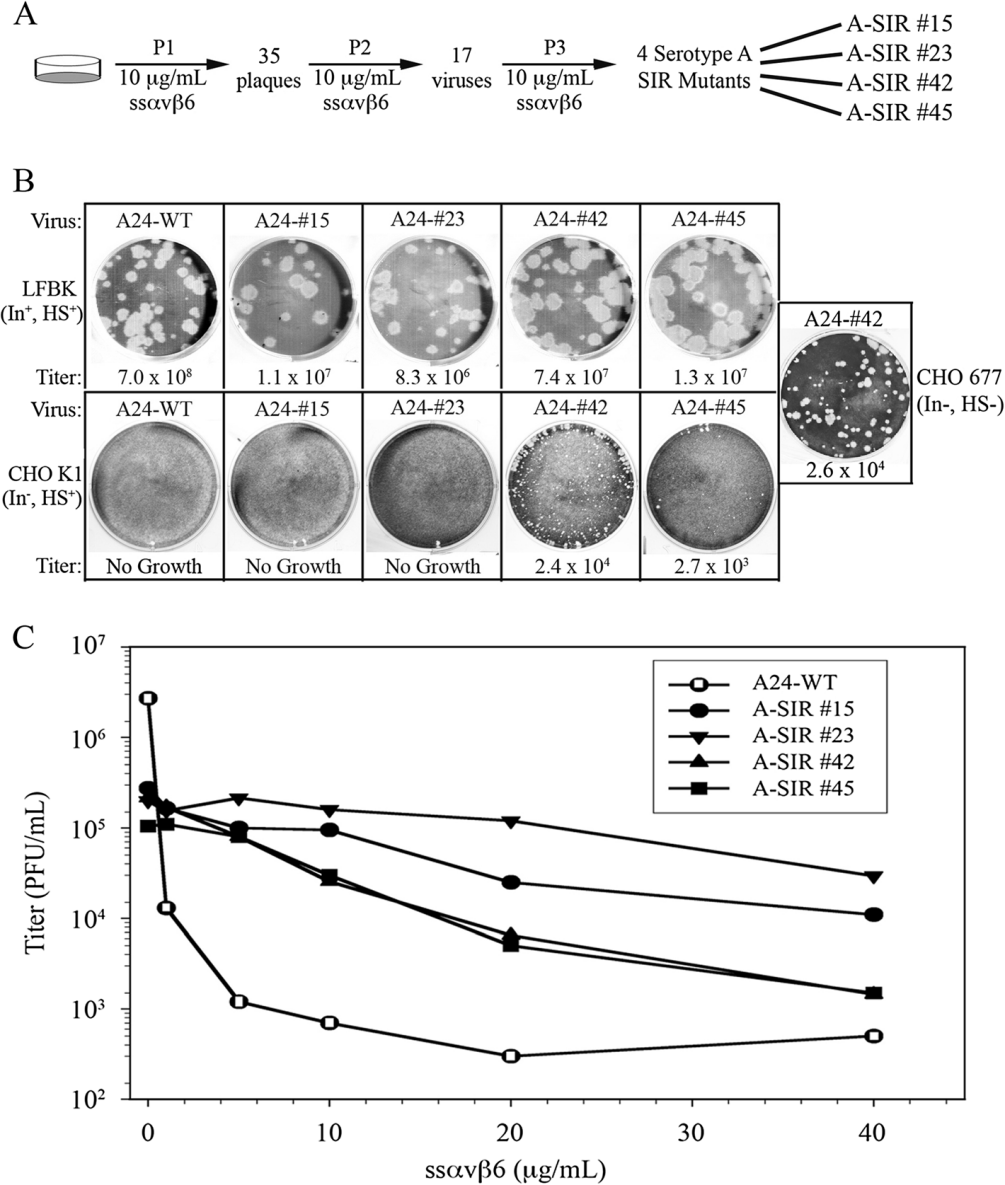
**A-SIR mutant generation. A.** Schematic of A-SIR mutant production. **B.** Comparison of the plaque morphology and titers achieved of A- SIRs relative to A24 Cruzeiro after pre-treatment and co-incubation with ssα_v_β_6_ on LFBK (top panel) and CHO K1 (bottom panel) cells for 24 h. SIR #42 was the only virus that amplified on CHO 677 cells (right box). Titers represent PFU/mL. **C.** WT and A-SIR viruses were co-incubated with increasing concentrations of ssα_v_β_6_ on LFBK monolayers for 24 h, and the calculated titers were subsequently plotted.

Wild-type (WT) FMDV A24 Cruzeiro was pre-incubated with 10 μg/mL ssα_v_β_6_ (sub-neutralizing concentration for A24 Cruzeiro, Additional file 1: Figure S1) at 37°C for 1 hour prior to application of the coated virus on LFBK cells. As depicted in Figure 2A, LFBK cells were infected with ssα_v_β_6_ coated WT FMDV A24 Cruzeiro at a MOI of 1 at 37°C for 24 hours (P1). Thirty-five plaques were isolated and used for a second round (P2) of infection with ssα_v_β_6_. Only 17 of the 35 infections demonstrated cytopathic effects (CPE) at 24 hpi. Isolates from 17 P2 infections were individually used for a third selection (P3) with ssα_v_β_6_. This resulted in only 4 productive infections derived from A24 Cruzeiro numbered: 15, 23, 42, and 45. The 4 soluble integrin resistant (SIR) serotype A (A-type) isolates (designated A-SIR #15, A-SIR #23, A-SIR #42, and A-SIR #45) were subject to a final selection with ssα_v_β_6_, where by 24 hpi A-type SIR isolates 15, 23, and 45 produced 100% CPE and isolate 42 produced 50% CPE.

After multiple passages in cell culture, FMDV has been shown to gain the ability to attach to host cells via HS [14,21-24,43]. To determine if the A-type SIR mutants adapted to use HS for entry, replication of these viruses on LFBK and Chinese hamster ovary (CHO) K1 cells was compared. In contrast to LFBK cells, CHO K1 cells express HS, but not α_v_β_1_, α_v_β_3_ and α_v_β_6_ (Table 1) [24,30-35]. Of note, CHO K1 cells express 2 RGD-binding integrins (α_5_β_1_ and α_v_β_5_), but neither function as FMDV receptors [7,10,30]. WT and A-SIR viruses successfully infected LFBK cells (Figure 2B, top panel). However, WT and A-SIRs #15 and #23 were unable to infect CHO K1 cells. In contrast, A-SIRs #42 and #45 productively infected CHO K1 cells (Figure 2B, bottom panel). Thus, we suggest that A-SIRs #42 and #45 circumvented ssα_v_β_6_ neutralization by expanding their receptor preference to include HS, as previously observed under other conditions with different FMDV serotypes [23,24].

The A-type SIR mutants diverged with respect to their plaque morphologies on different cell lines. WT and A-SIR mutants maintained relatively large plaque sizes on LFBK cells (Figure 2B, top panel). On CHO K1 monolayers, A-SIRs #42 and #45 showed reduced plaque size relative to LFBK cells (Figure 2B, lower panel). Comparative viral growth on LFBK and CHO K1 cells suggests some A-SIRs might utilize HS. To examine this further, the growth analysis was expanded to include the CHO 677 cell line (Figure 1, Table 1) [39,40,44]. In addition, the IBRS2 cell line with a unique receptor profile where FMDV infection is initiated via α_v_β_8_[9,16,42], was also examined (Table 1). IBRS2 cells were permissive to infection by WT and A-SIR viruses, growing to titers comparable to LFBK cells (Table 2). Interestingly, A-SIR #42 productively infected CHO 677 cells (Figure 2B, Table 2). SIR #42 grew to roughly equivalent titers on CHO 677 and CHO K1 cells (Figure 2B, Table 2). Viral growth on CHO 677 cells suggested this A-SIR mutant adapted to utilize an as yet unidentified and uncharacterized third FMDV receptor [21,41]. It was inferred that growth of A-SIR #45 on CHO K1 cells but not CHO 677 cells was indicative that this virus exploited HS. Different growth patterns for each A-SIR mutant on different cell lines also suggested these viruses are genetically distinct. These findings illustrated the rapid adaptability (after only 3 passages) of serotype A FMDV to overcome interference with host cell attachment.

**Table 2:** Growth comparison of A24 Cruzeiro WT and A-type SIRs on 6 cell lines

**Virus**	**LFBK**	**CHO K1**	**CHO 677**	**CHO 677 αvβ6**	**IBRS2**	**COS-1**
A24-WT	7 x 10^8^	-	-	6.3 x 10^7^	6 x 10^8^	-
A-SIR #15	1.1 x 10^7^	-	-	2.8 x 10^6^	4 x 10^7^	-
A-SIR #23	8.3 x 10^6^	-	-	1.8 x 10^6^	2 x 10^7^	-
A-SIR #42	7.4 x 10^7^	2.4 x 10^4^	2.6 x 10^4^	5.6 x 10^6^	6.5 x 10^7^	-
A-SIR #45	1.3 x 10^7^	2.7 x 10^3^	-	2 x 10^7^	1 x 10^7^	-

Numerical values represent virus titers in plaque forming units per milliliter (PFU/mL).

Dashes indicate no plaque detected at the lowest dilution of virus tested (1/10).

### A-SIR mutants exhibit reduced sensitivity to SI neutralization

Next, the relative sensitivities of A-SIR mutants to ssα_v_β_6_ neutralization were examined relative to their WT counterpart. Each virus was pre-incubated with ssα_v_β_6_ at gradually increasing concentrations (5, 10, 20, 30, and 40 μg/mL), applied to LFBK monolayers, and evaluated for reductions in virus titer. As shown in Figure 2C, the four A-SIR mutants could be divided into 2 classes with respect to sensitivity to neutralization by ssα_v_β_6_: a highly resistant (#15 and #23, Class I) and a moderately resistant class (#42 and #45, Class II). A-SIRs #15 and #23 grew to titers 200–1000 fold higher than WT with increasing ssα_v_β_6_, while mutants #42 and #45 exhibited titers 20–100 fold higher than WT (Figure 2C). Cumulatively, these findings reinforced the supposition that pre- and co-incubation of ssα_v_β_6_ acts as a selective pressure forcing FMDV particles to quickly select for resistance to the treatment.

### A-SIR mutants continue to utilize α_v_β_6_

**Figure 3:**
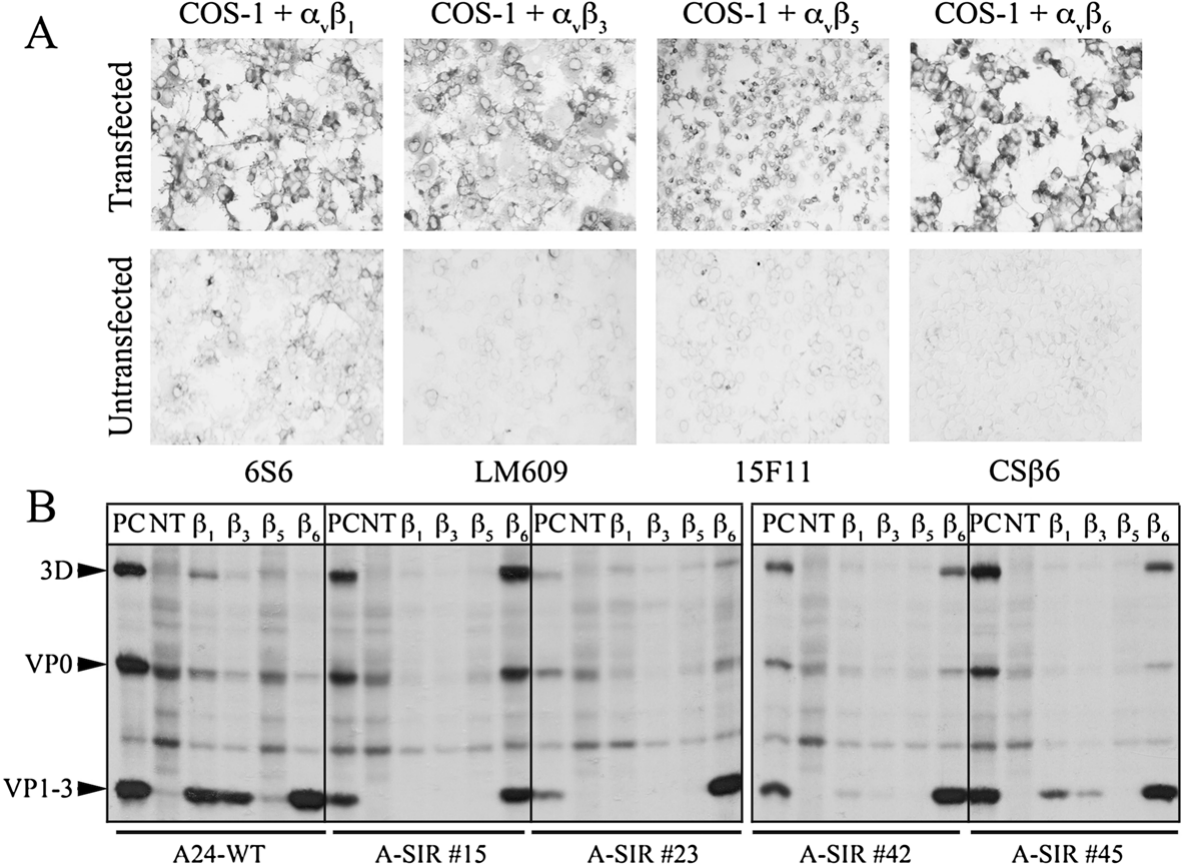
**A-SIR mutants maintain α**_**v**_**β**_**6 **_**affinity. A.** Expression of α_v_ and β subunits (β_1_, β_3_, β_5_, or β_6_) examined by immuno-histochemical staining using indicated antibodies. Untransfected cells probed as a negative control. **B.** RIP analysis of transfected cells infected with: A24 Cruzeiro and A-SIRs in the presence of ^35^S-methionine. NT, not transfected. PC, positive control: virus-infected LFBK cells.

Since the A-SIR mutants exhibited reduced sensitivity to ssα_v_β_6_ neutralization, we investigated whether they exhibit an altered integrin preference: shifting from α_v_β_6_ to another integrin or a non-integrin receptor. The capability of A-SIRs to infect cells expressing distinct integrins was tested using a previously established transient transfection-infection assay [45]. COS-1 cells do not support FMDV infection and lack integrins used by FMDV (Table 1) [7]. These cells were co-transfected with plasmids encoding the full-length α_v_-integrin subunit and 1 of 4 different full-length β subunits (β_1_, β_3_, β_5_, or β_6_). Expression was confirmed by immunocytochemical staining (Figure 3A). Of note, the antibody used to detect the α_v_β_1_ only recognizes the β_1_ subunit; as such, positive staining may be reflective of other β_1_ containing integrin heterodimers.

WT virus and A-SIR mutants were incubated on the cells transiently expressing integrin with ^35^S-methionine. After 24 hours, levels of viral protein synthesis were evaluated by radioimmunoprecipitation (RIP) (Figure 3B). Based on the sets where FMDV proteins (3D, VP0, VP1-3) were detected, it could be inferred as to which integrin heterodimers was/were used by WT and A-SIR viruses to gain entry to COS-1 cells for replication. Consistent with previous reports, viral protein synthesis was detected for A24 Cruzeiro WT in cells expressing α_v_β_1_, α_v_β_3_ and α_v_β_6_ (Figure 3B) [7,25]. A-SIRs #15 and #23 were only amplified on α_v_β_6_ expressing cells. A-SIRs #42 and #45 infected cells displaying α_v_β_1_ and α_v_β_6_. However, the signal for virus proteins was noticeably lower for A-SIR #42 and #45 relative to WT, though we cannot exclude the possibility that this was partially due to differences in transfection efficiencies. Besides WT, A-SIR #45 was the only other virus able to infect via α_v_β_3_. Interestingly, although resistant to neutralization by ssα_v_β_6_, all 4 A-SIR viruses replicated on α_v_β_6_ expressing COS-1 cells. Thus, it was inferred that all 4 A-SIR mutants maintained sufficient affinity for α_v_β_6_ to continue to infect cells via this receptor.

To reinforce the supposition that the A-SIR viruses retained the ability to bind α_v_β_6_, CHO 677 cells, which could not permit FMDV infection except for A-SIR #42, were transiently transfected with α_v_β_6_ as was done for COS-1 cells (data not shown). The expression of α_v_β_6_ restored infectivity of WT and A-SIR viruses, with titers within 0.5 to 1 log less than on LFBK cells (Table 2), with A-SIR #45 attaining a titer 1 log higher than both class I SIRs. Notably, A-SIR #42 achieved titers 2 logs higher than on untransfected CHO 677 cells, suggesting α_v_β_6_ is the preferred surface receptor for this virus.

### Amino acid substitutions detected in A-SIR mutants

**Figure 4:**
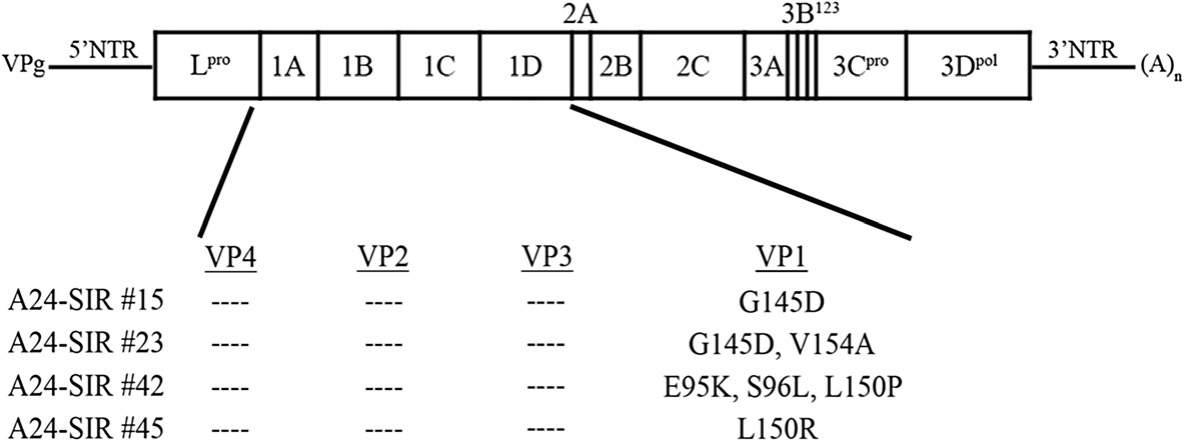
**Amino acid substitutions within the P1 region of A-SIR mutants.** Schematic of the FMDV genome summarizing the amino acid substitutions identified in the P1 region of A-SIR mutants after viral RNA from A24 Cruzeiro and A-SIRs was sequenced for amino acid substitutions in P1.

To further distinguish A-SIR viruses from their parental counterpart, A-SIR mutants were examined for alterations in their nucleotide and amino acid sequences relative to WT (Accession #AY593768). No amino acid substitutions were detected in VP4, VP2, or VP3, with a single silent mutation detected in VP3. Interestingly, the 2 A-type SIR classes exhibited 2 corresponding types of mutations in VP1 summarized in Table 3 and Figure 4. Class I A-SIRs that were highly resistant to ssα_v_β_6_ (#15 and #23) displayed a G145D amino acid substitution in the RGD motif considered essential for integrin interaction. The V154A substitution distinguished A-SIR #23 from #15. Class II A-type SIRs moderately resistant to ssα_v_β_6_ (#42 and #45) exhibited substitutions at the RGD + 4 position with L150P for #42 and L150R for #45. Moreover, A-SIR #42 displayed 2 additional amino acid alterations upstream of the RGD motif: E95K and S96L. Previous reports have described the helical segment immediately C-terminal to the RGD motif as contributing to the interaction between FMDV and its cognate integrin receptor, specifically, amino acid positions RGD + 1 and RGD + 4. The RGD + 1 and RGD + 4 side chains face out like those of the RGD motif and are frequently occupied by leucine residues [8,14,46-49]. While proline substitution at the RGD + 4 position was previously detected in the A5 Westerwald FRG/58 field isolate ( [50]b; [51]) and serially passaged populations of a serotype C FMDV strain [21], the arginine substitution at this position appears unprecedented. Cumulatively, the P1 sequences support the findings that 2 distinct classes of SIR mutants were derived from A24 Cruzeiro: where each class exhibited distinct forms of amino acid substitutions localized to the VP1 G-H loop.

**Table 3:** Sequence comparison of the P1 region of A24 Cruzeiro and A-SIRs

**Gene**	**A24 Cruzeiro WT**			**A-SIR #15 and #23 (Class I)**			**A-SIR #42 and #45 (Class II)**		
	**Nucleotide**^**a**^	**Codon**^**b**^	**Amino acid**^**c**^	**Nucleotide**^**a**^	**Codon**^**b**^	**Amino acid**^**c**^	**Nucleotide**^**a**^	**Codon**^**b**^	**Amino acid**^**c**^
VP2	C50	cgG	----	----	----	----	G50^45^	cgA	Silent
VP1	G6	acG	----	C6	acC	Silent	C6	acC	Silent
VP1	G283	Gaa	E95	----	----	----	A283^42^	Aaa	K95
VP1	C287	tCa	S96	----	----	----	T287^42^	tTa	L96
VP1	G434	gGc	G145	A434	gAc	D145	----	----	----
VP1	T449	cTc	L150	----	----	----	C449^42^	cCc^42^	P150^42^
							G449^45^	cGc^45^	R150^45^
VP1	T461	gTc	V154	C461^23^	gCc	A154^23^	----	----	----

^a^ Nucleotide differences between viruses followed by number indicating its position within the gene coding sequence.

^b^ Lowercase letters indicate bases shared by the 2 viruses and capital letters indicate bases differing between the viruses.

^c^ One-letter code of the encoded amino acid residues followed by number indicating residue position in the polypeptide.

P1 region of A24 Cruzeiro (Accession # AY596768) was compared against: Class I (#15 and #23) and Class II (#42 and #45) A-SIRs. Nucleotide changes relative to WT were indicated and codon positions. Silent mutations and amino acid substitutions are indicated. A-SIR mutant numerical designations were superscripted.

### Structural predictions of A-SIR mutant modifications

**Figure 5:**
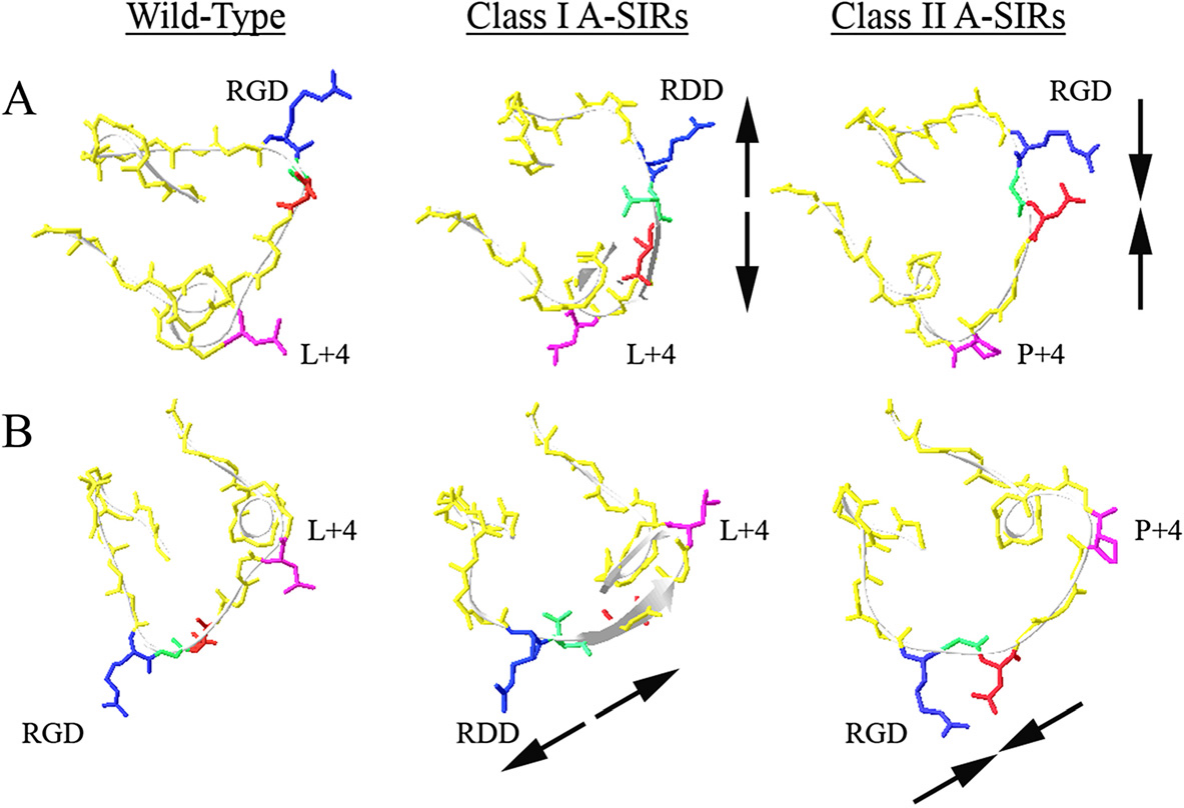
**Structural prediction of the effect of the A-SIR amino acid substitutions on the G-H loop.** Depicted are 2 angles (**A** and **B**) of models of the VP1 G-H loop of A24 Cruzeiro and representative class I and class II A-SIRs. RGD motif is indicated, and residues are colored: arginine (R), blue; glycine (G), green; aspartic acid (D), red. RGD + 4 position (L/P + 4) is violet. Black arrows indicate altered directionality of the amino acid side chains in A-SIR RGD/RDD sites.

After defining the amino acid changes within VP1 in the A-SIR mutants (Figure 4), we explored how those changes might affect the overall structure of the receptor-binding site in the G-H loop. The VP1 crystal structure has been solved for FMDV O1/BFS 1860/UK/67 (Accession 1FOD) [52]. Using the coordinates of 1FOD as a template, homology structures were generated of WT A24 Cruzeiro VP1 as well as those of the 4 A-SIR mutants using the Geno3D algorithm [53]. For each virus, 10 different “best fit” homology models were generated for VP1. Representative images of the G-H loop region were given particular scrutiny (Figure 5).

The RGD to RDD substitution detected in class I A-SIR mutants #15 and #23 placed a bulky negatively charged residue into the center of the integrin binding site, which can be seen projecting outward in the homology structures generated. The introduction of D145 “spread out” the adjacent side chains within the RGD motif, increasing the distance between the R144 and D146 side chains relative to WT (Figure 5). In all 10 of the predicted tertiary structures, the amino acid alteration failed to effect any significant change in the backbone structure of this region of VP1.

The previously undetected L150R substitution at the RGD + 4 position of class II A-SIR #45 replaces a linear non-polar amino acid side chain with a positively charged residue. Interestingly, Geno3D failed to predict a consensus structure for the A-SIR #45 G-H loop. Class II A-SIR mutant #42 exhibited a L150P substitution at RGD + 4, which was previously identified in FMDV A5 Westerwald FRG/58 [50,51]. The homology model of the A-SIR #42 G-H loop revealed the proline residue introducing a kink that “compressed” the RGD motif with the three side chains in much closer proximity than WT (Figure 5). This structural prediction was essentially the opposite of what was produced for the class I A-SIR mutants.

In addition to L150P, A-SIR #42 also displayed 2 substitutions upstream of the RGD: E95K and S96L, which are proximal to the VP1-VP3 interface. Using Geno3D, ribbon diagrams were generated of capsid protomers from WT and A-SIRs to determine if the protein interface was disrupted (Additional file 2: Figure S2). A short helical domain observed at the VP1-VP3 interface in the WT sequence was notably absent with the E95K/S96L substitution, replaced with a loop structure. With L150P previously detected in A5 Westerwald, E95K/S96L may be more significant to the adaptation of A_SIR #42. We concluded that the mutations elucidated in the A-SIR mutants produce hypothetical structural alterations likely to affect cellular recognition.

### O1 Campos derived SIR mutants circumvent ssα_v_β_6_ by a different mechanism

**Figure 6:**
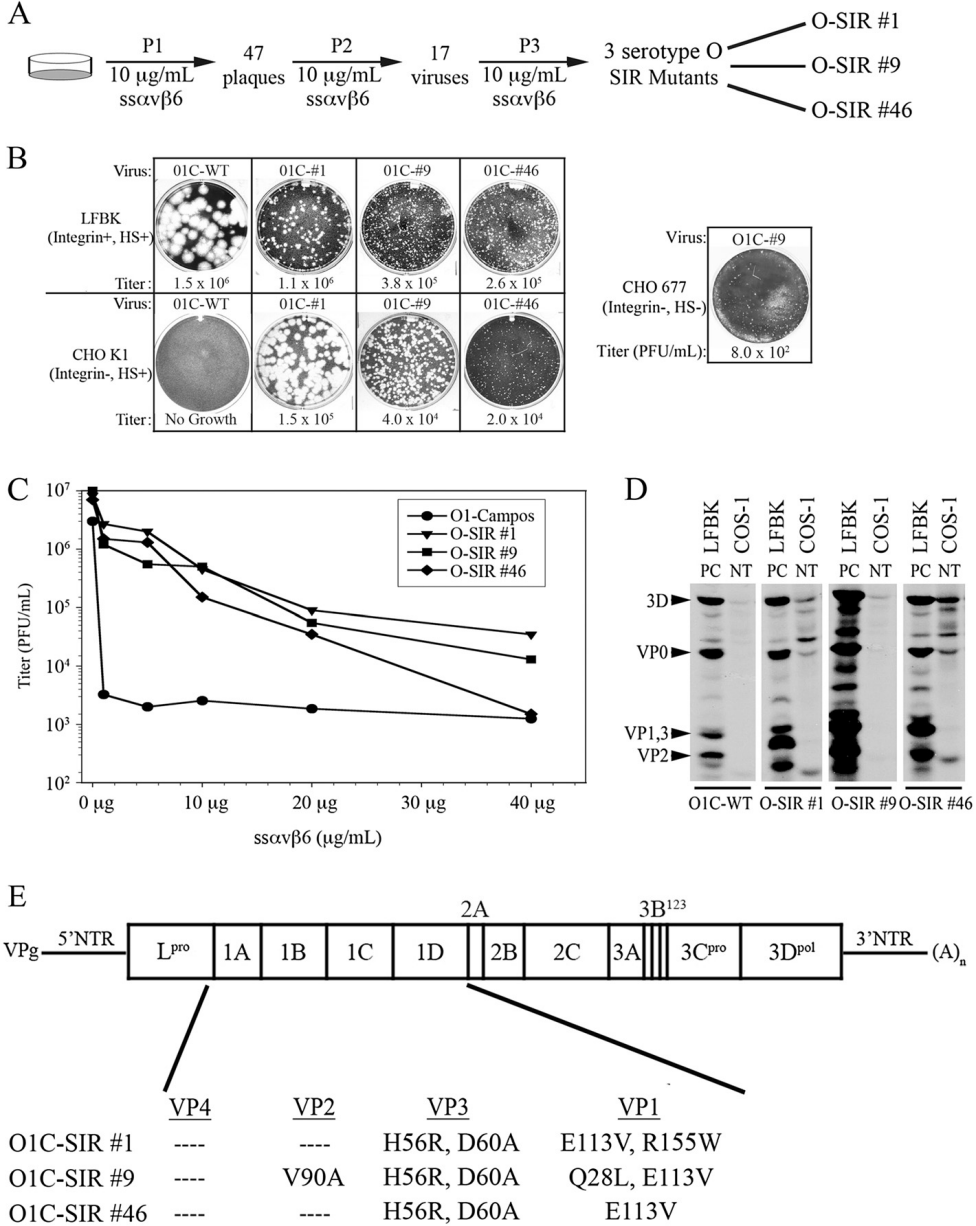
**O1 Campos derived SIR mutants exhibit distinctive alterations. A.** Schematic of O1 Campos derived O-SIR mutant production. **B.** Comparison of the growth characteristics of O-SIRs relative to O1 Campos after ssα_v_β_6_ co-incubation on LFBK and CHO K1 cells. O-SIR #9 was the only virus that amplified on CHO 677 cells. Titers represent PFU/mL. **C.** WT and O-SIR viruses were co-incubated with ssα_v_β_6_ on LFBK monolayers for 24 h, and the calculated titers were subsequently plotted. **D.** RIP analysis of untransfected LFBK and COS-1 cells infected with: O1 Campos and O-SIRs in the presence of ^35^S-methionine. NT, not transfected (COS-1). PC, positive control (LFBK). **E.** FMDV genome depicting amino acid substitutions identified in the P1 region of O-SIR mutants.

A-SIR mutants derived from A24 Cruzeiro overcame the selective pressure introduced by ssα_v_β_6_ by compensatory mutations targeted to the RGD motif or the RGD + 4 position within the VP1 G-H loop. In an effort to determine if a similar strategy would be exploited by a different FMDV serotype, the experiment was repeated whereby O1 Campos was pre-treated and passaged three times in the continued presence of 10 μg/mL ssα_v_β_6_ (also a sub-neutralizing concentration for O1 Campos, data not shown). Three O-type SIR viruses were recovered designated O-SIR #1, O-SIR #9, and O-SIR #46 (Figure 6A). All three O-SIRs produced similar small plaque forming units on LFBK cells relative to their WT progenitor (Figure 6B, top panel). Unlike the WT virus, the O-SIRs productively infected CHO K1 cells (Figure 6B, bottom panel), achieving titers approximately 1 log lower than on LFBK cells (Table 4). This suggested that the O-SIRs adapted to the presence of ssα_v_β_6_ by selecting an affinity for HS, which represents a well-characterized strategy employed by serotype O FMDV [22,23,54]. Each O-SIR mutant produced distinct plaque morphologies on CHO K1 cells; with large plaques for O-SIR #1, medium-sized plaques for O-SIR #9, and small pinprick plaques for O-SIR #46 (Figure 6B, bottom panel). Interestingly, like the A-type SIR mutant #42, O-SIR #9 could replicate, albeit to a limited titer, on CHO 677 cells (Figure 6B, right panel and Table 4). When CHO 677 cells transiently expressing α_v_β_6_ were substituted in this assay, infectivity was restored for all O-SIR mutants and O1 Campos WT, with titers equivalent to or exceeding those on LFBK cells (Table 4). This particular finding was consistent with what was observed for the A-SIR viruses, where affinity for α_v_β_6_ was maintained. Additionally, both O1 Campos and the O-SIR mutants demonstrated titers 1–2 logs higher on IBRS2 cells relative to LFBK cells (Table 4), which suggested that these viruses infect via α_v_β_8_ with greater efficiency.

**Table 4:** Growth comparison of O1 Campos WT and O-type SIRs on 6 cell lines

**Virus**	**LFBK**	**CHO K1**	**CHO 677**	**CHO 677- αvβ6**	**IBRS2**	**COS-1**
O1C-WT	1.5 x 10^6^	-	-	4 x 10^8^	8 x 10^7^	-
O-SIR #1	1.1 x 10^6^	1.5 x 10^5^	-	9 x 10^5^	1.9 x 10^8^	*
O-SIR #9	3.8 x 10^5^	4 x 10^4^	8.0 x 10^2^	1.5 x 10^6^	5 x 10^8^	*
O-SIR #46	2.6 x 10^5^	2 x 10^4^	-	1.5 x 10^6^	1.6 x 10^8^	*

Numerical values represent virus titers in plaque forming units per milliliter (PFU/mL).

Dashes indicate no plaque detected at the lowest dilution of virus tested (1/10).

Asterisks indicate limited abortive infection without plaque formation, indicated by thinning of the cell monolayer.

Further analysis revealed that all three O-SIRs were uniformly 2 logs more resistant to neutralization by ssα_v_β_6_ up to 20 μg of ssα_v_β_6_ (Figure 6C). However, while this resistance was maintained by O-SIR #1 and O-SIR #9 above 20 μg ssα_v_β_6_, O-SIR #46 was inhibited by ssα_v_β_6_ at concentrations above 20 μg. Initial examinations of the reliance of the O-SIRs on different integrin heterodimers revealed the presence of FMDV proteins in untransfected COS-1 cells infected with the O-SIR mutants, similar to the pattern observed for O-SIR infected LFBK cells (Figure 6D). No increase in viral protein synthesis was observed for cells transiently expressing integrins (data not shown). Like CHO K1 cells, COS-1 cells express HS, thus it was inferred that the O-SIR mutants had circumvented ssα_v_β_6_ neutralization by adapting to utilize HS for host cell attachment.

Given the disparities in plaque morphology and sensitivity to soluble integrin neutralization, it was expected that the O-type SIR mutants would be genetically distinct. In contrast to A-SIRs, compensatory mutations were not confined to VP1, but were also identified within VP3 (Figure 6E). The mutations detected in the O-SIRs were consistent with those previously found in HS-adapted serotype O FMDV passaged multiple times in cell culture with a signature H56R substitution in VP3 [22,23,54]. An additional D60A substitution in VP3 and an E113V substitution in the VP1 G-H loop were found in all O-SIR viruses. O-SIRs were distinguished from each other by 2 unique amino acid substitutions: R155W in the VP1 G-H loop of O-SIR #1 and Q28L in VP1 and V90A in VP2 of O-SIR #9. Notably, the conserved RGD motif and RGD + 4 amino acid positions were unchanged.

Cumulatively, the development and characterization of the A-SIR and O-SIR FMDV mutants and the evolution of the virus-host interaction under the selective pressure of soluble integrin treatment was determined in the present study.

## Discussion

Distinct lineages of RNA viruses are frequently referred to as “quasi-species” due to their inherent mutability [47,55]. Their rapid adaptability stems from the lack of “proofreading” function in the RNA-dependent RNA polymerases responsible for amplification of RNA viral genomes. The poliovirus RNA polymerase delivers approximately 1 error in 2200 bases, which is significant for a virus genome of approximately 7500 nucleotides [56]. Constant variation with each duplicated RNA viral population represents a stumbling block in the development of anti-viral therapies. A previous study explored using soluble receptor treatments to impair FMDV infection [25,49]. Here, we investigated the effect of sub-neutralizing levels of soluble secreted α_v_β_6_ (ssα_v_β_6_) on FMDV replication in cells and documented the rapid emergence of resistant FMDV populations, how they circumvented the selective pressure of ssα_v_β_6_, and the strategies deployed as they adapted.

Previous studies have investigated the effect of multiple passaging in cell culture on FMDV acquisition of alternative receptors, attenuated phenotypes, and for the dispensability of the RGD motif [21,49,57]. For instance, following 100 passages of a serotype C FMDV (C-S8c1) in cell culture, the virus was able to enter cells by an integrin-independent manner and multiple mutations were identified in the variant genomes [21]. For serotype A12 virus, it has been shown that mutations obtained during adaptation of field (bovine) isolates to cell culture localize downstream of the RGD and those mutations appear to alter the affinity for the integrin receptor [49].

Here, four SI resistant (SIR) FMD viruses derived from A24 Cruzeiro and three derived from O1 Campos were rapidly selected after only 3 rounds of cell culture selection in the continued presence of ssα_v_β_6_. The A-SIR mutants could be separated into 2 classes that were either highly or moderately resistant to neutralization by ssα_v_β_6_. Highly resistant A-SIRs (Class I) did not select HS as a secondary receptor and maintained α_v_β_6_ affinity. These variants displayed a G145D substitution in the highly conserved VP1 RGD motif. The moderately resistant A-SIRs (Class II) also retained α_v_β_6_ affinity and appeared to either utilize HS or an as yet unidentified and uncharacterized third FMDV receptor. Moreover, similar to A24 Cruzeiro, all 4 A-SIR mutants were amplified on IBRS2 cells, indicating that the A-SIRs have maintained the ability to infect and replicate on cells expressing an abundance of α_v_β_8_, which can also function as a FMDV receptor [9,16,42].

### Class I A-SIR Mutants

A-SIR mutants appear to have circumvented ssα_v_β_6_ treatment by 2 different routes. Substitution of an aspartic acid (G145D) in the RGD motif (Figure 4) rendered A-SIR #15 and 23 (Class I) highly resistant to ssα_v_β_6_ (Figure 2C) while paradoxically maintaining use of α_v_β_6_ for infection (Figure 3B). Notably, class I A-SIRs did not adapt to utilize HS. Interestingly, this RDD mutation in the cell receptor binding site has been detected in a field strain of Asia1 virus (Asia1/JS/CHA/05) after just 2 passages, one *in vivo* and one *in vitro*[57]. RDD Asia1 variants showed no change in their ability to replicate in established cell lines, nor did it alter clinical onset of disease in animals tested [57]. While the environmental pressure that selected for RDD Asia1 variants is unknown, it suggests that this mutation might be a natural adaptation mechanism that could manifest across different FMDV serotypes. This hypothesis is supported by the observation that natural outbreaks of serotype A FMDV featuring the RDD amino acid substitution have occurred in Argentina on two occasions: A25-Arg/59 (GenBank #AY593769) and A25-Arg/61 (GenBank #AY593789).

The likely reason for the selection of a modified RGD was also investigated using homology models of the VP1 G-H loop structure and position of the RDD side chains (Figure 5). The data showed an additional negative charge provided by G145D widened the gap between the side chains of R144 and D146. Potentially, the widening of the RDD side chains or the introduction of an additional negatively charged side chain might diminish the affinity of the motif for the integrin by sterically complicating the binding of ssα_v_β_6_, which is not anchored to a membrane, to the virus particle during pre-treatment in solution. Correspondingly, by reducing the affinity but not ablating the interaction with α_v_β_6_ (Figure 3B, Table 2), unoccupied RDD sites on the virus particle after pre-treatment and subsequent co-incubation with ssα_v_β_6_ likely allow infection of host cells via α_v_β_6_.

### Class II A-SIR Mutants

In contrast, moderately resistant Class II A-SIRs left the RGD intact, but substituted a key amino acid at the RGD + 4 position [8,14,46-48], with the conserved leucine replaced with proline for A-SIR #42 and arginine for A-SIR #45. L150P was previously identified in FMDV A5 Westerwald FRG/58 [50,51] as well as a multiply passaged serotype C FMDV [21,58]. However, L150R appears unprecedented. Interestingly, while both viruses replicated in CHO K1 cells, only A-SIR #42 infected CHO 677 cells. However, both class II A-SIRs exhibited preference for α_v_β_6_ over alternative surface molecules (Figure 2, Table 2). Given L150P was previously detected in the WT field strain A5 Westerwald [51], it will be interesting to explore in the future whether this amino acid alteration was solely responsible for shifting receptor tropism to allow infection of cells devoid of FMDV integrin receptors and HS [21,30,38,41]. However, two additional mutations detected in VP1, E95K and S96L, might also contribute or be exclusively responsible for the A-SIR #42 phenotype.

Homology modeling was also employed to illustrate how Class II amino acid substitutions affected both the G-H loop (Figure 5) and the capsid protomer (Additional file 2: Figure S2). In contrast to Class I A-SIRs, L150P appears to compress the side chains projecting from the RGD motif, where R144 and D146 are in closer proximity than in A24 Cruzeiro or Class I A-SIRs (Figure 5). The unique E95K/S96L substitutions localized to the VP1-VP3 interface, potentially altering capsid protomer stability (Additional file 2: Figure S2). Modeling algorithms failed to generate a consensus structure for the G-H loop of A-SIR #45, where multiple potential side chain angles for L150R altered the shape and presentation of the RGD motif (data not shown).

### O-SIR mutants

When the approach used to generate the A-SIRs was applied to O1 Campos, this resulted in three distinctive O-SIR mutants that bind to HS and that carried a signature H56R mutation. This particular mutation has been observed in attenuated HS-adapted FMDVs [22,23,54]. Similar to their A-type counterparts, the O-SIRs also could be amplified on IBRS2 cells suggesting that the selective pressure of co-incubation with ssα_v_β_6_ did not abrogate the affinity for α_v_β_8_. However, unlike A24 Cruzeiro and the A-SIRs, O1 Campos and the O-type SIR mutants amplified 2–3 logs more in IBRS2 cells relative to LFBK cells, thus it could be inferred that serotype O FMDV may have a preference for α_v_β_8_ as a receptor. As such it would be intriguing to explore the effects of successive passages of O1 Campos in the presence of ssα_v_β_8_ in the future. Lastly, it is interesting to note that the mutations accumulated by the O-SIRs were not in the VP1 RGD motif, but rather peripheral to it and at locations in VP3, separate from the primary site of attachment to the host cell.

## Conclusions

Together the results of this study have shed light on the plasticity of FMDV serotype A and O interactions with its primary cognate receptor: α_v_β_6_. Amino acid substitutions detected in SIR mutants were rapidly selected to overcome soluble receptor neutralization. Thus, the tolerance of the VP1 G-H loop to amino acid substitutions plays an essential role in cell receptor adaptability of FMDV. Interestingly, mutations identified in the current study have been also identified in FMDV field isolates causing outbreaks suggesting that similar selective pressures may exist in the natural host environment. The mode and relative speed with which FMDV was able to adapt to a selective pressure, represented here by ssα_v_β_6_, highlights both the evolutionary advantage of highly mutable RNA viruses and the challenges of designing effective antiviral therapies against these pathogens.

## Materials and methods

### Materials

Fugene-6 was purchased from Roche (Nutley, NJ). Mouse monoclonal anti-β_3_ integrin (ITGβ3) was purchased from Abcam (Cambridge, MA). Rabbit polyclonal anti-β_6_ integrin (ITGβ6) was purchased from Sigma (St. Louis, MO). Mouse monoclonal anti-β_1_ integrin (6S6), anti-α_v_β_3_ integrin (LM609), anti-α_v_β_5_ integrin (15 F11), and anti-β_6_ integrin (CSβ6) was purchased from Millipore (Billerica, MA). Rabbit polyclonal anti-RHA was purchased from Bethyl Laboratories (Montgomery, TX).

### Cells, viruses, and plasmids

IBRS2, COS-1, and 293a cell lines purchased from American Tissue Collection Company (ATCC; Manassas, VA) were cultured in Dulbecco’s minimal eagle medium (DMEM) with 10% fetal bovine serum (FBS) at 37°C with 5% CO_2_. LFBK cell line was previously described [29], and cultured in DMEM with 10% FBS at 37°C with 5% CO_2_. CHO K1 and 677 cell lines were acquired from Dr. Jeffrey Esko [39] and cultured in Ham’s MEM (Gibco) with 10% FBS at 37°C with 5% CO_2_. FMDV A24 Cruzeiro field strain was derived from pA24-Cru [59]. FMDV O1 Campos field strain was previously described [24].

### SI production

Expression and purification of ssα_v_β_3_ and ssα_v_β_6_ was previously described [25]. Plasmids encoding ectodomains of integrin subunits (α_v_, β_3_, and β_6_) were transfected using Fugene-6 (Roche) per manufacturer’s instructions. Stable expression was selected by incubating with G418 (α_v_ gene sub-cloned into pcDNA3.1-G418) and zeomycin (β_3_ and β_6_ gene sub-cloned into pcDNA3.1-Zeo). Supernatants were collected and concentrated 10-fold using Centricon Plus-70 filter devices (Millipore, Billerica, MA). Subsequent protein concentration measured between 125–250 μg/mL.

### Radio-immunoprecipitation (RIP)

Virus-infected cells grown overnight in the presence of ^35^S-methionine were lysed with 1% Triton X100. Aliquots of each sample were precipitated with 20% trichloroacetic acid (TCA) to determine the counts per minute (cpm). Lysates were mixed with Protein A/G agarose beads, tumbled 15 minutes, centrifuged at 1500 rpm 10 minutes, and the supernatants collected. Recovered supernatant was tumbled with indicated antibodies (6S6, LM609, 15 F11, and CSβ6) 1 hour at 4°C. The mixture was tumbled overnight at 4°C with fresh Protein A/G agarose. Afterwards, the agarose was washed 3 times with NET/NP40 buffer (NaCl, EDTA, Tris, Nonidet-P40). Finally, the agarose was boiled in sample buffer without β-mercaptoethanol, pelleted, the supernatants separated by SDS-PAGE, and the gel examined by autoradiography.

### Transient transfection-infection assay

Assay was conducted as previously described [45]. Two sets of COS-1 or CHO 677 cells were transfected with 2 plasmids: one encoding full-length α_v_-integrin subunit and the other encoding 1 of 4 different full-length β subunits (β_1_, β_3_, β_5_, or β_6_). The first sets were examined by immuno-histochemical staining to confirm expression, using antibodies: 6S6 (α_v_β_1_), LM609 (α_v_β_3_), 15 F11 (α_v_β_5_), and CSβ6 (α_v_β_6_). 6S6 binds the β_1_ subunit and COS-1 cells express α_5_β_1_, which is not a cellular receptor for FMDV [11,12,21]. The other sets were infected with WT and SIR viruses at a MOI of 1 in ^35^S-methionine containing media. Subsequently, virus-infected cell lysates were examined by RIP for virus-specific bands: 3D, VP0, and VP1-3.

### Virus titer assay

One hour post-adsorption, the inoculum was removed, and cells washed in a mild acid solution followed by virus growth media (VGM, DMEM containing L-glutamine). VGM was then added and cells incubated 24 hours at 37°C. Afterwards, virus-infected cells were harvested and titers determined by plaque assay as previously described [60]. Plates were fixed, stained with crystal violet (0.3% in Histochoice; Amresco, Solon, OH), and plaques counted. Values calculated for number of plaque-forming units (PFUs) per milliliter (mL) were plotted using Microsoft Excel (Microsoft Corporation, Redmond, WA). Assays were performed in triplicate.

### Western blot

Protein samples were separated by Nu-PAGE® pre-cast gel system (Invitrogen), and electro-blotted onto nitrocellulose (Sigma). After blocking with 5% milk, proteins were detected with indicated primary integrin antibodies (anti-β_3_, Abcam and anti-β_6_, Sigma) followed by HRP-conjugated goat-anti-mouse or goat-anti-rabbit antibodies (Bethyl Laboratories), respectively. Cellular tubulin, employed as a loading control, was detected with HRP-conjugated anti-tubulin-α (Abcam, Cambridge, MA). HRP was reacted with WestDura SuperSignal chemiluminescent reagent (Pierce) and visualized on X-ray film (X-Omat; Kodak, N.Y., USA).

### Sequencing of the FMDV P1 Region

The P1 region in twenty FMDV isolates were sequenced from PCR product with sequencing primers providing at least 3X coverage across 3,000 base pairs. PCR products were purified with QIAQuick spin columns (Qiagen) in accordance with the manufacturer's instructions. Sequencing reaction mixtures (10 μl) contained 2.5 μM primer, 20 ng of PCR product, and 0.75 μl of Big Dye (Applied Biosystems) in molecular biology-grade water. The sequence cycling conditions were 30 s of pre-incubation at 85°C; 25 cycles of 10 s at 96°C, 5 s at 50°C, and 4 min at 60°C; and a 10-min 60°C final extension. The sequencing reaction mixtures were purified with Agencourt’s CleanSEQ system in accordance with the manufacturer's instructions (Beckman Coulter).

### Structural analysis

Amino acid sequences of SIRs were used to construct homology models of the G-H loop and capsid protomers using the Geno3D algorithm [53]. Ten most likely structures were generated using a solved structure as a template. Two X-ray crystal structures for the major immunogenic site of FMDV designated 1FOD [52] and 1ZBE [61] in the Protein Data Bank (PDB) were selected as templates. Hypothetical structures were examined using DeepView [62,63], and a consensus structure for each sequence was selected.

## Competing interests

The authors declare that they have no competing interests.

## Authors’ contributions

PL: evaluated the integrin expression on the tested cell lines, organized the sequencing data and performed the sequencing alignments, constructed the structural models, and drafted the manuscript. ML: produced the FMDV SIR mutants, evaluated SIR plaque morphologies and growth kinetics on indicated cell lines, and performed the radio-immunoprecipitation analysis. BB: co-conceived the study and participated in its design and implementation. ER: co-conceived the study and participated in its design, implementation, and coordination. All authors have read and approved the final version of the manuscript.

## Supplementary Material

Additional file 1 **Figure S1.** Soluble integrin neutralization assay. Titration experiment performed where A24 Cruzeiro was pre-incubated with gradually increasing amounts of ssαvβ6 to determine a suitable sub-neutralizing soluble receptor concentration. Click here for file

Additional file 2 **Figure S2.** Structural prediction of the effect of the A-SIR amino acid substitutions on the capsid protomer. Depicted are 3 ribbon models of the FMDV capsid protomer (excluding VP4) of A24 Cruzeiro (A) and class I (B) and class II (C) A-SIRs. Black arrows indicate the RGD motif and the RGD + 4 position. Blue arrows indicate the VP1-VP3 interface. VP1 is yellow, VP2 is blue, and VP3 is red. Click here for file
