# Supplementary figures and images for: Examination of soluble integrin resistant mutants of foot-and-mouth disease virus
Source: Virol J. 2013 Jan 2;10:2. doi: 10.1186/1743-422X-10-2 (PMC3547720; doi:10.1186/1743-422X-10-2)

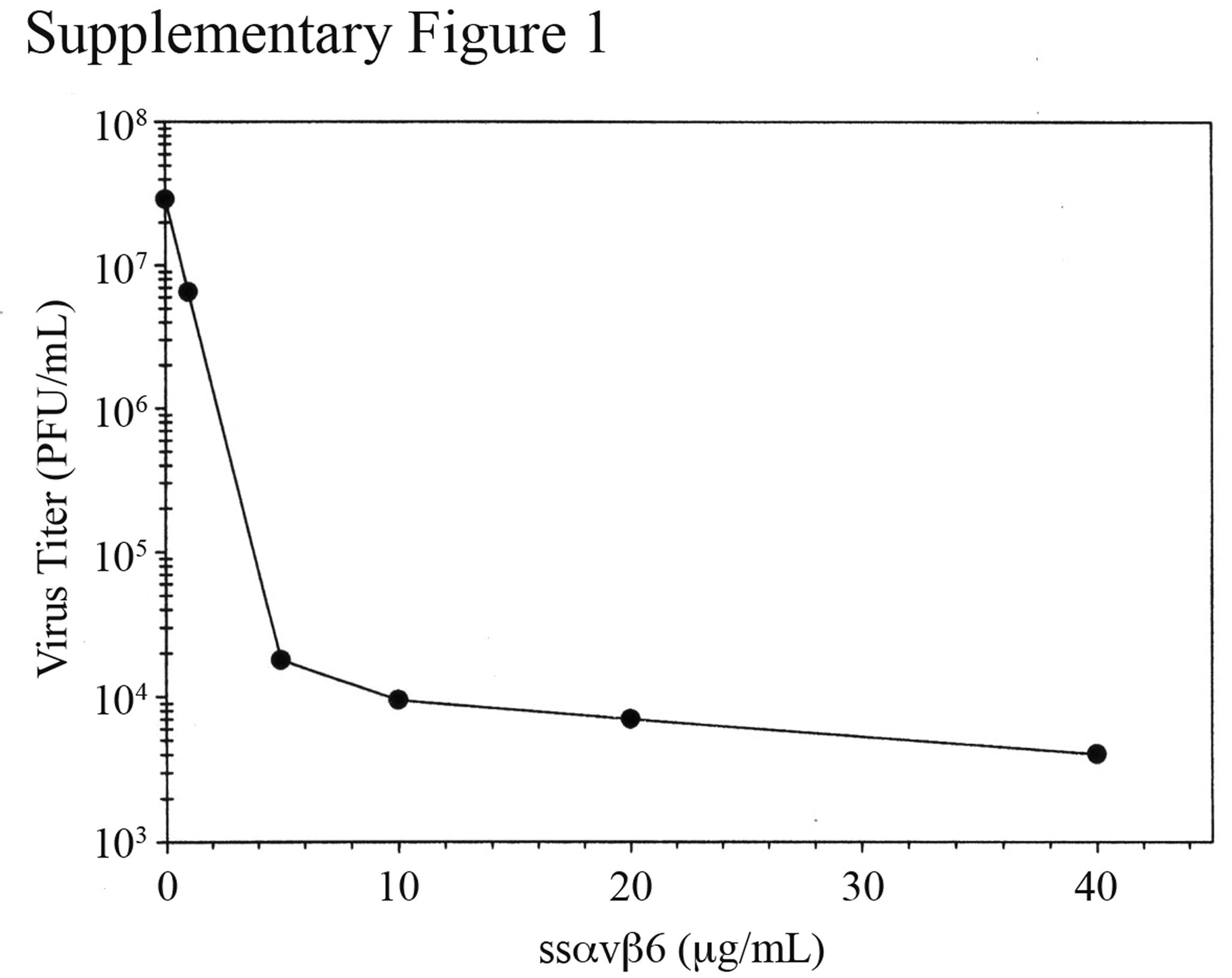

Supplement: Additional file 1 — Figure S1. Soluble integrin neutralization assay. Titration experiment performed where A24 Cruzeiro was pre-incubated with gradually increasing amounts of ssαvβ6 to determine a suitable sub-neutralizing soluble receptor concentration. [file 1743-422X-10-2-S1.jpeg]

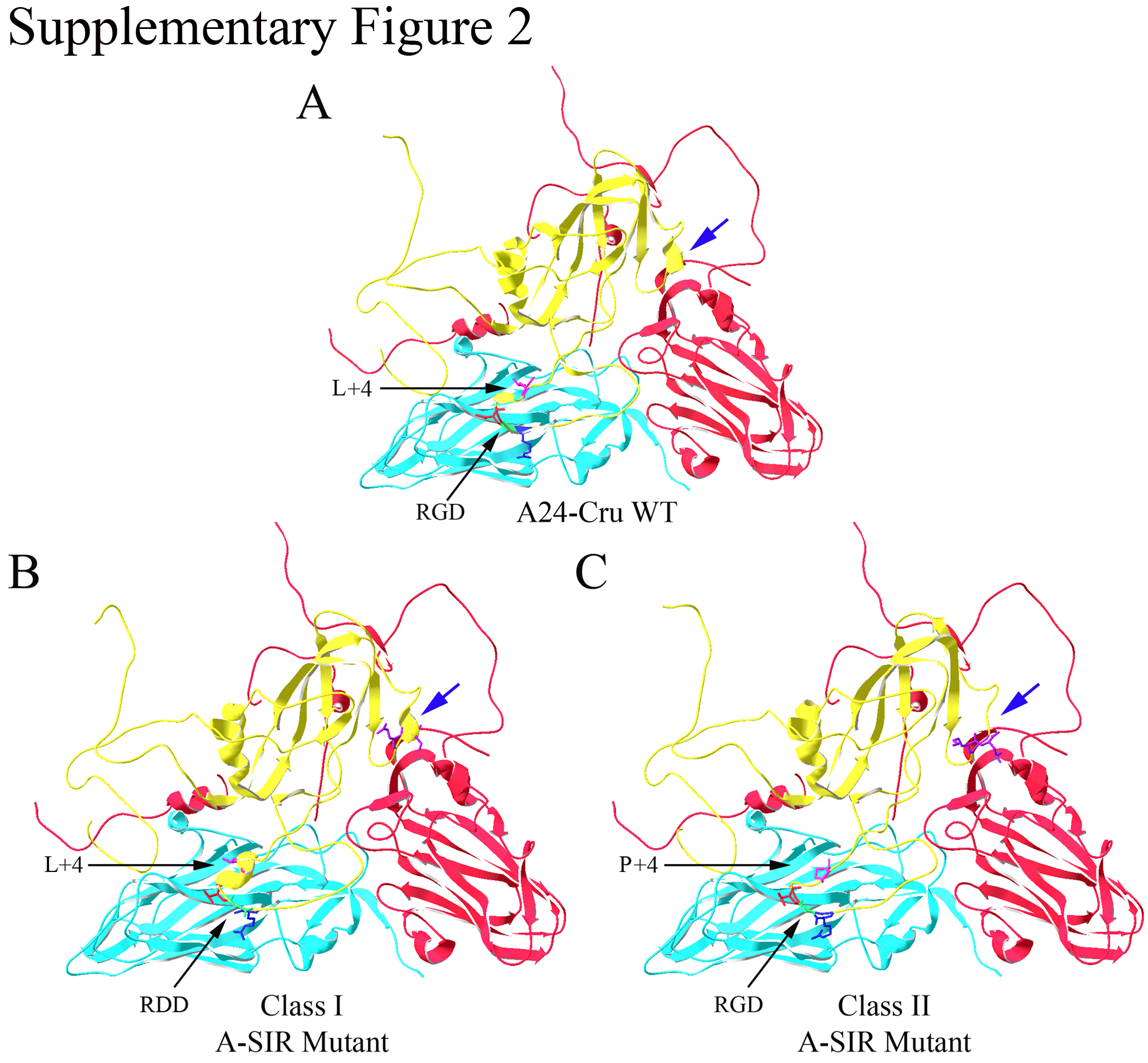

Supplement: Additional file 2 — Figure S2. Structural prediction of the effect of the A-SIR amino acid substitutions on the capsid protomer. Depicted are 3 ribbon models of the FMDV capsid protomer (excluding VP4) of A24 Cruzeiro (A) and class I (B) and class II (C) A-SIRs. Black arrows indicate the RGD motif and the RGD + 4 position. Blue arrows indicate the VP1-VP3 interface. VP1 is yellow, VP2 is blue, and VP3 is red. [file 1743-422X-10-2-S2.jpeg]
